# Chlorophyll-Mediated Changes in the Redox Status of Pancreatic Cancer Cells Are Associated with Its Anticancer Effects

**DOI:** 10.1155/2018/4069167

**Published:** 2018-07-02

**Authors:** Kateřina Vaňková, Ivana Marková, Jana Jašprová, Aleš Dvořák, Iva Subhanová, Jaroslav Zelenka, Iva Novosádová, Jan Rasl, Tomáš Vomastek, Roman Sobotka, Lucie Muchová, Libor Vítek

**Affiliations:** ^1^Institute of Medical Biochemistry and Laboratory Diagnostics, 1st Faculty of Medicine, Charles University, Prague, Czech Republic; ^2^Department of Biochemistry and Microbiology, University of Chemistry and Technology Prague, Prague, Czech Republic; ^3^Institute of Microbiology, Academy of Sciences of the Czech Republic, Prague, Czech Republic; ^4^4th Department of Internal Medicine, 1st Faculty of Medicine, Charles University, Prague, Czech Republic

## Abstract

Nutritional factors which exhibit antioxidant properties, such as those contained in green plants, may be protective against cancer. Chlorophyll and other tetrapyrrolic compounds which are structurally related to heme and bilirubin (a bile pigment with antioxidant activity) are among those molecules which are purportedly responsible for these effects. Therefore, the aim of our study was to assess both the antiproliferative and antioxidative effects of chlorophylls (chlorophyll *a*/*b*, chlorophyllin, and pheophytin *a*) in experimental pancreatic cancer. Chlorophylls have been shown to produce antiproliferative effects in pancreatic cancer cell lines (PaTu-8902, MiaPaCa-2, and BxPC-3) in a dose-dependent manner (10–125 *μ*mol/L). Chlorophylls also have been observed to inhibit heme oxygenase (HMOX) mRNA expression and HMOX enzymatic activity, substantially affecting the redox environment of pancreatic cancer cells, including the production of mitochondrial/whole-cell reactive oxygen species, and alter the ratio of reduced-to-oxidized glutathione. Importantly, chlorophyll-mediated suppression of pancreatic cancer cell viability has been replicated in *in vivo* experiments, where the administration of chlorophyll *a* resulted in the significant reduction of pancreatic tumor size in xenotransplanted nude mice. In conclusion, this data suggests that chlorophyll-mediated changes on the redox status of pancreatic cancer cells might be responsible for their antiproliferative and anticancer effects and thus contribute to the decreased incidence of cancer among individuals who consume green vegetables.

## 1. Introduction

**Figure 1:**
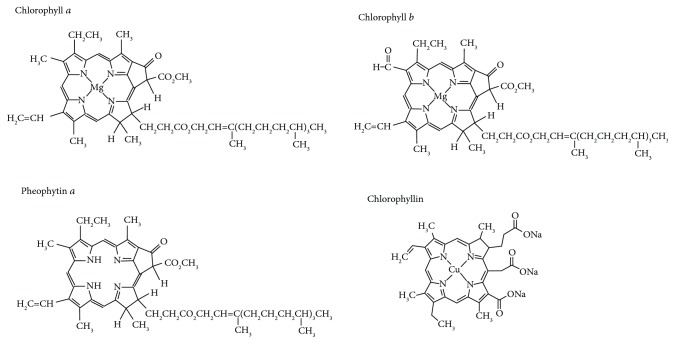
Structures of chlorophylls.

Bioactive nutritional factors, such as those contained in green plants, may be involved in the protection of individuals from the development of cancer [1]. In fact, a noticeable cancer protection effect has been observed when the dietary intake of green vegetables is increased [2]. Chlorophyll and other tetrapyrrolic compounds, which are structurally related to bilirubin (the potent antioxidant bile pigment) [3], are among the important candidate molecules which are considered to be responsible for this protective effect [1, 4]. Chlorophyll, a phytol-esterified magnesium porphyrin, is one of the most abundant biomolecules on Earth [5]. Several chlorophyll species occur in nature, with chlorophyll *a* and chlorophyll *b* being the most important [4, 6]. In addition, chlorophyllin, a more polar semisynthetic chlorophyll, is used as an additive in the food industry and thus is rather abundant in parts of the human food chain [4]. This characteristic is also true for pheophytin *a*, a metal-free derivative dietary chlorophyll metabolite that is prominent in both fresh and processed foods, as well as in dietary supplements [4] (Figure 1).

Due to a variety of biological effects including antigenotoxicity [7], mutagen trapping [8], immunomodulation, and antioxidant and apoptotic activities (for review, see [4]), chlorophylls have been proposed as important dietary chemopreventive agents [4]. Chlorophylls tightly bind to a wide range of carcinogens in the intestinal lumen which prevents their absorption in the body [1]. This mechanism was observed in a clinical study that was conducted in China, in which chlorophyllin supplementation substantially reduced aflatoxin availability in exposed subjects [9]. Similarly, in a clinical study conducted in the Netherlands, patients whose dietary intake of chlorophyll was decreased experienced an increase in the incidence of colon cancer [10]. In the latter study, the authors proposed that chlorophyll was instrumental in blocking the potential carcinogenic effects of heme in the intestinal lumen [11]. Although generally chlorophylls are assumed to be nonabsorbable from the intestinal lumen, it is possible that a significant fraction of chlorophylls as well as their derivatives might be bioavailable and thus active even in peripheral tissues [4].

Heme oxygenase 1 (HMOX1) and biliverdin reductase (BLVRA) are key enzymes in heme catabolism and bilirubin formation, whose systemic concentrations have a direct and negative relationship on the prevalence of cancer [12]. HMOX1 is an important cytoprotective and antioxidant enzyme, not only in humans [13] but also in the plant kingdom [14], although its potential role in carcinogenesis has yet to be resolved [15]. Numerous epidemiological studies have shown that an increase in *HMOX1* expression is associated with a decrease in the incidence of cancer [16]. On the other hand, *HMOX1* expression has been found to interfere with anticancer treatments against pancreatic cancer [17]. In addition, it has been proposed that BLVRA plays a role in the promotion of carcinogenesis [18]. In fact, increased *BLVRA* expression has been observed in patients with hepatocellular cancer [19] and in breast and lung cancer cell lines [20].

Since there is very little published data that describes the antiproliferative effects of chlorophylls, the aim of our study was to assess these effects and determine if they can be mediated via HMOX1 modulation and/or redox signaling pathways.

## 2. Materials and Methods

### 2.1. Chemicals

*Spirulina (Arthrospira) platensis* was purchased from Martin Bauer GmbH (Vestenbergsgreuth, Germany). Hemin was obtained from Frontier Scientific (Logan, UT, USA), and chlorophyllin, chlorophyll *a*, chlorophyll *b*, crystal violet, thiazolyl blue tetrazolium bromide (MTT), dimethylsulfoxide (DMSO), cell culture media, and supplements were purchased from Sigma-Aldrich (St. Louis, MO, USA). Acetone, methanol, dioxane, acetonitrile, and glutaraldehyde (25% solution) used in all experiments were obtained from Penta (Prague, Czech Republic). Methanol and acetonitrile were obtained from Merck (Darmstadt, Germany).

### 2.2. Preparation of Pheophytin *a*

For the preparation of pheophytin *a*, a cyanobacterium *Synechocystis* PCC 6803 (ATCC, Manassas, VA, USA) culture (4 L) was harvested. The cells were broken using glass beads in 20 mM K-phosphate buffer (pH 7.8). The membrane fraction from the cells was separated from soluble proteins by high-speed centrifugation (65,000 ×g, 20 min). The pelleted membranes were lyophilized overnight. The pigments were extracted from the dried membranes by 2 × 2.5 mL of methanol and separated using an Agilent-1200 HPLC system (Agilent, Santa Clara, CA, USA).

The separation was carried out on a reversed-phase column (Luna C8, 5 *μ*m, 250 × 10 mm; Phenomenex) using 35% methanol and 15% acetonitrile in 0.25 M pyridine (solvent A) and 20% methanol and 20% acetone in acetonitrile (solvent B). The pigments were eluted via a linear gradient of solvent B (30–95% in 25 min) followed by 95% of solvent B at a flow rate of 2 mL min^−1^ at 40°C. The peak representing chlorophyll *a* was collected, and the resulting solution (8 mL) acidified (pH ~3) with acetic acid to convert chlorophyll *a* to pheophytin *a*. In order to remove pyridine and other polar impurities, the solution was mixed in a 1 : 1 ratio with 0.25 M NaCl, followed by an addition of 10 mL of hexan. After mixing, the solution was incubated for 15 min at room temperature and the upper hexan phase containing pheophytin *a* was taken. The hexan was evaporated on a rotary evaporator, and the dried pheophytin was then dissolved in DMSO.

The purity of all pigments (hemin, chlorophyllin, chlorophyll *a*, chlorophyll *b*, and pheophytin *a*) used in this study was checked chromatographically by HPLC.

### 2.3. Cell Cultures

The following human pancreatic adenocarcinoma cell lines were used for *in vitro* studies: PaTu-8902 (DSMZ, Braunschweig, Germany), MiaPaCa-2, and BxPC-3 (ATCC). All cell lines were maintained in a humidified atmosphere (5% CO_2_ at 37°C) in DMEM medium containing 10% fetal bovine serum (PaTu-8902, MiaPaCa-2) or RPMI (BxPC-3). The cell lines were authenticated at ATCC by STR profiling before distribution and also reauthenticated at the end of the study by an external laboratory (Generi Biotech, Hradec Kralove, Czech Republic).

### 2.4. Cell Viability Assays

The viability of each tumor cell line was determined using the MTT assay. After 24 h of incubation of the cell lines with the tested compounds, the culture media were replaced with fresh media containing MTT (1 mg/mL). After additional 2 h of incubation, the resulting formazan complex was dissolved in DMSO. Absorbance was then measured at 540 nm using a Sunrise ELISA reader, and the data was assessed using the Magelan-6 program (Tecan, Austria).

### 2.5. Heme Oxygenase Activity Determination

PaTu-8902 human pancreatic cancer cells were incubated for 24 h with the experimental compounds. After incubation, the cells were washed with phosphate-buffered saline (PBS, 0.1 M, pH 7.4), harvested, centrifuged, and resuspended in PBS. The cell suspension, stored on ice, was disrupted by sonication and incubated for 15 min at 37°C with methemalbumin (50 *μ*M) and NADPH (1.5 mM). HMOX activity was determined by measuring carbon monoxide production using gas chromatography, as previously described [21]. Cells treated with hemin were used as a positive control for HMOX activity induction.

### 2.6. RNA Isolation and Real-Time PCR

After four hours of incubation with experimental compounds, mRNA from PaTu-8902 human pancreatic cancer cells was isolated using the PerfectPure RNA Cell Kit (5Prime, Hamburg, Germany), according to the manufacturer's instructions. Isolated mRNA was then dissolved in nuclease-free water, and its quality and concentration were measured on a Nanodrop 2000 spectrophotometer (Thermo Fisher Scientific, Franklin, MA, USA). Reverse transcription of mRNA to cDNA was performed using a High-Capacity cDNA kit (5Prime, Hamburg, Germany). The *HMOX1*, *BLVRA*, and *HPRT* primer sequences (Table 1) were used, as previously described [22].

To determine the relative expression level of *HMOX1* and *BLVRA*, *HPRT* expression level was measured acting as an internal control. Two reference genes (*HPRT* and *GAPD*) were selected which were observed as the most stable among four constant genes (*HPRT*, *GAPD*, *18S RNA*, and *UBC*), based on the analyses of the DNA in peripheral blood leukocytes from 10 healthy human controls using geNORM 3.5 (https://genorm.cmgg.be/ (accessed 2018 May 27)). Based upon similar expression levels as in the target genes, *HPRT* was chosen as the more appropriate control gene. The relative change was calculated as 2^−ΔΔCT^. qPCR was performed in a 20 *μ*L reaction volume, containing 4 *μ*L of cDNA template which was diluted 5 times from the completed reverse transcription (RT) reaction, 1x SYBR Green Master Mix (Applied Biosystems, Foster City, CA, USA), and 200 nM forward and reverse primers. All RT-PCR were conducted in 96-well optical plates and run in triplicate on an ABI PRISM 7500 Sequence Detector System (Applied Biosystems). The cycling conditions included polymerase activation at 95°C for 10 min, followed by 40 cycles of 95°C for 15 s and 60°C for 60 s [22].

### 2.7. Determination of Mitochondrial Superoxide Production

The production of superoxide anion in the mitochondrial matrix was calculated as the time-dependent increase in fluorescence intensity of a selective MitoSOX dye (Life Technologies, Pleasanton, CA, USA) in cells that had been incubated with the potential therapeutics. The final determination was carried out by flow cytometry (BD LSRII, BD Biosciences, San Jose, CA, USA). After 24 h incubation with the therapeutics of interest, MitoSOX was added to the cells for 15 minutes. Before each measurement, the cells were treated with 10 *μ*M rotenone in order to increase the production of mitochondrial superoxide. The change in fluorescence was measured at one-minute intervals for a period of 16 min.

### 2.8. Determination of the GSH/GSSG Ratio

The PaTu-8902 cell samples were harvested, pelleted, and washed with PBS. After centrifugation of the cell suspension (200 ×g, 5 min), the PBS was removed and the pellets were lysed in a solution of acetonitrile : water (62.5 : 37.5, *v*/*v*) and properly mixed. Lysates were centrifuged at 20,000 ×g for 20 min at 4°C; then, the glutathione-containing supernatant was collected, snap frozen in liquid nitrogen, and stored in liquid nitrogen until analysis. Samples were analyzed by capillary electrophoresis (Agilent 7100) equipped with a polyimide-coated fused-silica capillary (60 cm × 50 *μ*m). The separation was done in 40 mM phosphate buffer (pH 7) (Sigma-Aldrich) at a voltage of 30 kV and measured at a wavelength of 195 nm. Reduced and oxidized glutathione was detected as separate peaks and identified by comparison to commercial standards (Sigma-Aldrich). The percentage of glutathione disulfide (GSSG) was calculated from the areas of the individual peaks of glutathione (GSH) and GSSG.

### 2.9. Determination of H_2_O_2_ Production

The production of hydrogen peroxide from the PaTu-8902 cells was measured as the oxidation rate of the fluorogenic Amplex red indicator (Life Technologies) in the presence of horseradish peroxidase (HRP, type VI-A, Sigma-Aldrich), as previously described [23]. Briefly, cells were preincubated for 24 h with the pigments, then washed with PBS and resuspended in a quartz cuvette. Amplex red (5 *μ*M) and HRP (10 U/mL) were then added to the suspension, and the fluorescence (565/585) was measured over a 10-minute period spectrofluorimetrically (RF-5301-PC, Shimadzu Corporation, Kyoto, Japan).

### 2.10. Determination of Antioxidant Capacity

The peroxyl radical-scavenging capacity was measured in solutions of different concentrations of the various chlorophylls, based on the proportion of antioxidant consumption relative to Trolox, as previously described [24].

### 2.11. Determination of Reductive Carboxylation

The degree of reductive carboxylation and glutaminolysis was evaluated as described previously [25]. The cells were incubated until an 80% confluence is reached after which the medium was replaced with fresh medium containing ^13^C-L-glutamine (Cambridge Isotope Laboratories Inc., Cambridge, MA, USA) and incubated at 37°C for additional six hours. Finally, the cells were harvested, and the pellets were extracted with water/methanol/chloroform (1 : 1 : 2, *v*/*v*/*v*; Penta, Czech Republic) and centrifuged at 1000 ×g for 10 min. The upper polar phase was transferred to a glass vial and lyophilized overnight. The analytes were derivatized with pyridine/N-(trimethylsilyl)acetamide/chlorotrimethylsilane (9 : 3 : 1, *v*/*v*/*v*; Sigma-Aldrich) at 65°C for one hour. The derivatized samples were injected directly into a gas chromatography/mass spectrometry device (GC-MS, GC 6890N, MD 5973, Agilent) equipped with crossbond diphenyl dimethyl polysiloxane (Restek Column). A temperature gradient (10°C per minute) was used during GC analysis, and selected ions were monitored on the mass detector. The activity of reductive carboxylation was determined by the rate of ^13^C incorporation from glutamine to both citrate (*m*/*z* 273 and 274) and malate (*m*/*z* 335 and 336). Moreover, 2-hydroxyglutarate (*m*/*z* 349 and 350) synthesis was observed. The rate of reductive carboxylation was calculated as the percentage of ^13^C-metabolite (citrate, malate), along with the rate of 2-hydroxyglutarate synthesis. Lactate concentration (pmol of lactate per million cells) was measured according to a similar protocol with two exceptions: (1) the cells were treated for 12 h before harvesting and replenishing the culture with fresh nonlabelled medium (without ^13^C-labelled glutamine) and (2) the internal standard (oxalate, *m*/*z* 190, Sigma-Aldrich) was added before the analytic preparation of the pellets.

### 2.12. Determination of ERK and AKT Activation

Cells grown at 50% confluency were lysed in RIPA buffer which was supplemented with phosphatase and protease inhibitors (Protease-Inhibitor Mix G and Phosphatase-Inhibitor-Mix I, Serva, Heidelberg, Germany). The cell lysates were clarified by centrifugation, separated by SDS-PAGE, blotted to 0.45 *μ*m nitrocellulose membranes, blocked in 5% nonfat milk in PBS, and probed with primary antibodies. Fluorescent labeled anti-rabbit IRDye 700 and anti-mouse IRDye 800 secondary antibodies (LI-COR Biosciences, Lincoln, NE, USA) were used for visualization using an Odyssey infrared imaging system (LI-COR Biosciences). The rabbit phospho-p44/42 MAPK (Erk1/2), rabbit phospho-Akt (Ser473), mouse p44/42 MAPK (Erk1/2), and rabbit AKT primary antibodies were obtained from Cell Signaling Technology (Danvers, MA, USA). The mouse p120RasGAP antibody was obtained from ECM Biosciences LLC (Versailles, KY, USA).

### 2.13. In Vivo Experiments

*In vivo* studies were performed on athymic nu/nu mice (Charles River Wiga, Sulzfeld, Germany) xenotransplanted subcutaneously with human pancreatic adenocarcinoma PaTu-8902 cells (10^7^ cells/mouse; control group (*n* = 7) and treated group (*n* = 6)). After reaching the basal tumor size (0.15 to 0.2 cm^3^, 7 days after xenotransplantation), chlorophyll *a* treatment was initiated by intragastric administration once daily via a gastric tube (1.5 mg/kg body weight/day) for 30 days. Chlorophyll *a* was dissolved in a small amount of 96% ethanol and diluted to the appropriate volume with clean vegetable oil. The control group was treated with pure vegetable oil. Tumor sizes were assessed by the measurement of the two greatest perpendicular diameters of subcutaneous tumors which were measured every three days with a caliper [26]. All aspects of these animal studies, as well as all of the protocols, met the accepted criteria for care and experimental use of laboratory animals and were approved by the Animal Research Committee of the 1st Faculty of Medicine, Charles University, Prague.

### 2.14. Statistical Analyses

The differences between variables were evaluated by the Mann–Whitney rank sum test. Group mean differences in tumor size were measured by repeated measures analysis of variance (RM ANOVA) with Holm-Šidák post hoc testing when *p* values were significant. Linear regression analyses were used to compare the effects of *A. platensis* treatment on total peroxyl-scavenging activity *in vitro*. Depending on their normality, the data are presented as the mean ± SD or the median and 25–75% range. Differences were considered statistically significant when *p* values were less than 0.05.

## 3. Results

### 3.1. The Effect of Chlorophylls on Pancreatic Cancer Cell Viability

All of the chlorophylls tested decreased cell viability in a dose-dependent manner within a concentration range between 10 and 125 *μ*mol/L (Table 2). In general, a biologically relevant effect was consistently observed from 50 *μ*M concentrations in chlorophylls *a* and *b*, whereas the effects of pheophytin *a* and chlorophyllin were marginal or absent (Table 2). The pancreatic cancer cell line most sensitive to the antiproliferative effects of chlorophylls was PaTu-8902 (Table 2) which was used for further studies.

### 3.2. The Effect of Chlorophylls on HMOX1 and BLVRA mRNA Expression in Human Pancreatic Cancer

**Figure 2:**
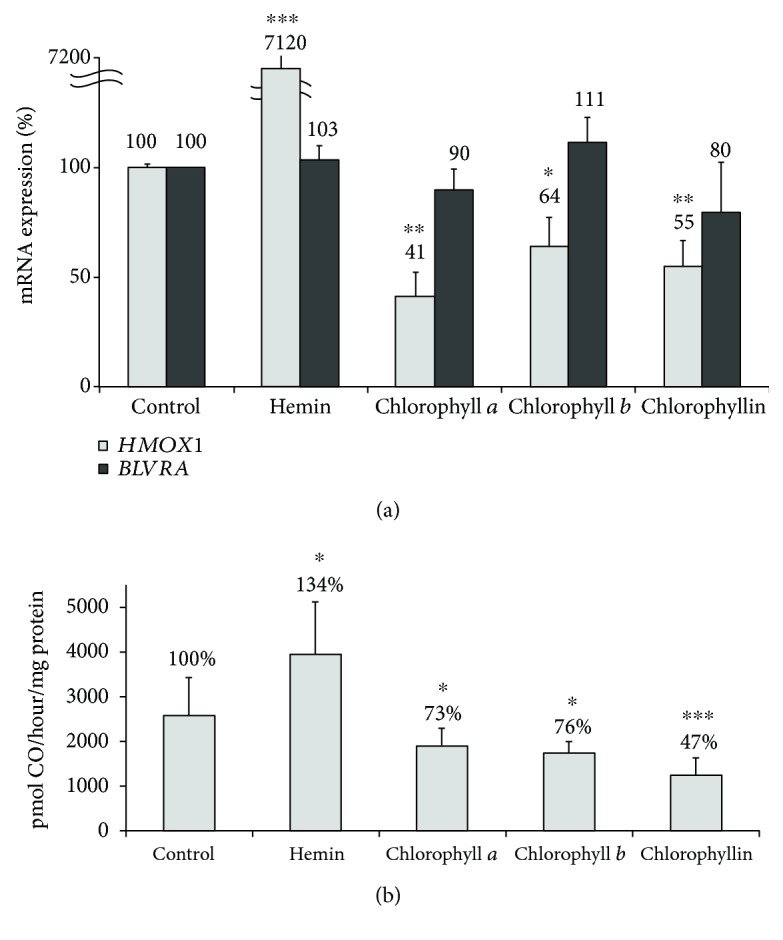
The effect of chlorophylls on *HMOX1* and *BLVRA* mRNA expression (a) and HMOX activity (b) in PaTu-8902 human pancreatic cancer cells. HMOX1: heme oxygenase 1; BLVRA: biliverdin reductase A. *c* = 10 *μ*mol/L for chlorophyll *a* and chlorophyll *b*; *c* = 30 *μ*mol/L for chlorophyllin and hemin. Hemin was used as a positive control for HMOX activity induction. *n* = 8 in each group. ^∗^*p* < 0.05, ^∗∗^*p* < 0.005, ^∗∗∗^*p* < 0.001.

Due to the structural similarity between chlorophylls and human bile pigments and the immense role of the heme catabolic pathway in the modulation of oxidative stress and redox biology, we studied the effect of chlorophylls on *HMOX1* and *BLVRA* mRNA expression in PaTu-8902 human pancreatic cancer cells. While no effect on *BLVRA* mRNA expression was observed upon treatment with any of the chlorophylls tested, *HMOX1* mRNA expression was significantly downregulated by all chlorophylls (Figure 2(a)). Accordingly, HMOX activity in pancreatic cancer cells was also significantly inhibited by all chlorophylls (*p* < 0.001) with chlorophyllin emerging as the most potent HMOX inhibitor (to 44 ± 10%, *p* < 0.001 (Figure 2(b))).

### 3.3. The Effect of Chlorophylls on the Parameters of Oxidative Stress and Redox Status of Human PaTu-8902 Pancreatic Cancer Cells

**Figure 3:**
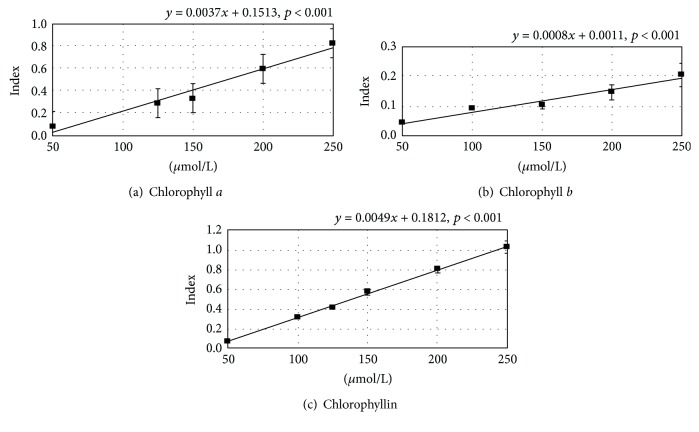
The effect of chlorophylls on peroxyl radical-scavenging capacity. Index = fluorescence intensity relative to Trolox. *n* = 5 in each group.

Next, we examined the antioxidant activity of chlorophylls. Using an *in vitro* assay, all chlorophylls tested showed the capacity to scavenge peroxyl radicals in a dose-dependent manner with chlorophyllin being the most potent (Figure 3).

**Figure 4:**
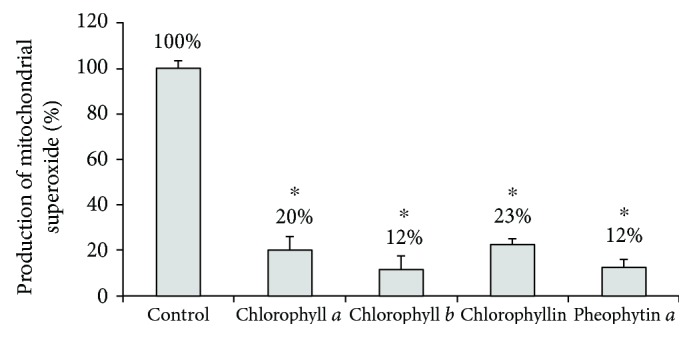
The effect of chlorophylls on mitochondrial superoxide production in PaTu-8902 human pancreatic cancer cells. *c* = 10 *μ*mol/L for chlorophyll *a*, chlorophyll *b*, and pheophytin *a*; *c* = 30 *μ*mol/L for chlorophyllin, ^∗^*p* < 0.001, *n* = 4 in each group.

**Figure 5:**
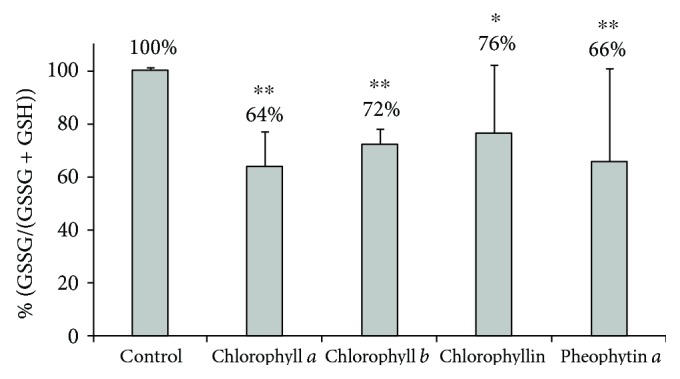
The effect of chlorophylls on intracellular glutathione status in PaTu-8902 human pancreatic cancer cells. *c* = 10 *μ*mol/L for chlorophyll *a*, chlorophyll *b*, and pheophytin *a*; *c* = 30 *μ*mol/L for chlorophyllin, *n* = 5 in each group, ^∗^*p* < 0.05, ^∗∗^*p* < 0.005. The *y*-axis represents GSSG/(GSSG + GSH) relative to control (%).

**Figure 6:**
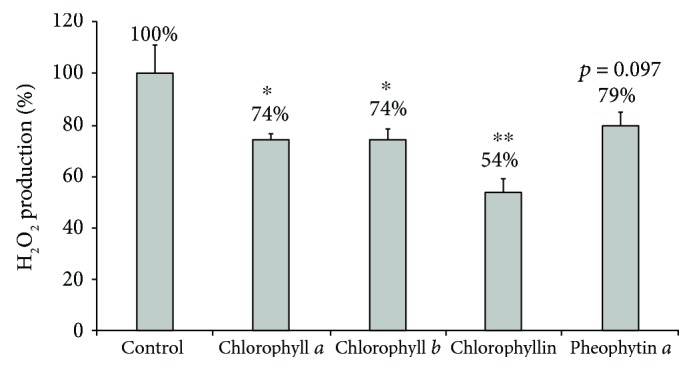
The effect of chlorophylls on whole-cell hydrogen peroxide production by PaTu-8902 human pancreatic cancer cells. *c* = 50 *μ*mol/L, ^∗^*p* < 0.05, ^∗∗^*p* < 0.01, *n* = 4 in each group.

Based on these results, we then investigated whether chlorophylls can reduce the mitochondrial production of superoxide in treated pancreatic cancer cells. In fact, in all of the tested chlorophylls, concentrations as low as 10 *μ*M substantially decreased mitochondrial superoxide production in pancreatic cancer cells (Figure 4). Additionally, when exposed to any of the tested chlorophylls, increased proportions of reduced glutathione were observed in the pancreatic cancer cells, suggesting an attenuated production of intracellular oxidants (Figure 5). Furthermore, hydrogen peroxide production by human pancreatic cancer cells was also significantly affected by higher concentrations of the tested chlorophylls (50 *μ*mol/L (Figure 6)) while lower concentrations showed no effect (10 *μ*mol/L, data not shown).

### 3.4. The Effect of Chlorophylls on Reductive Carboxylation and Glutaminolysis of Human PaTu-8902 Pancreatic Cancer Cells

Due to the apparent inhibitory effects of chlorophylls on the production of mitochondrial reactive oxygen species (ROS), we were interested in whether chlorophylls could modulate mitochondrial glutaminolysis and reductive carboxylation which contribute to mitochondrial antioxidant protection [25, 27]. In those studies, the exposure of human PaTu-8902 pancreatic cancer cells to chlorophyll *a*, chlorophyll *b*, pheophytin *a*, and chlorophyllin (50 *μ*mol/L for all pigments) did not lead to any significant changes in ^13^C incorporation from glutamine into 2-hydroxyglutarate and *α*-ketoglutarate (both markers of active glutaminolysis), nor into citrate and malate (both markers of reductive carboxylation). Additionally, no change in the production of lactate was observed, suggesting an undisturbed balance between mitochondrial respiration and glycolysis (data not shown).

### 3.5. The Effect of Chlorophylls on ERK and AKT Activation in Human PaTu-8902 Pancreatic Cancer Cells

**Figure 7:**
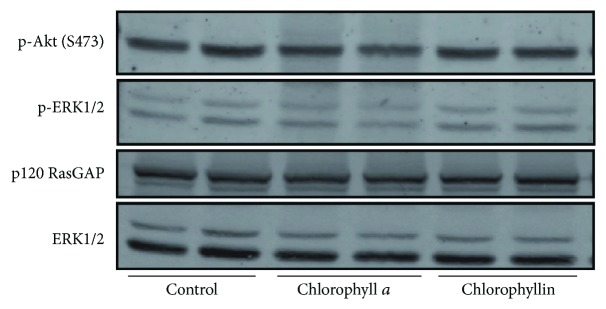
The effect of chlorophylls on the AKT and ERK activation in PaTu-8902 human pancreatic cancer cells. PaTu-8902 pancreatic cancer cells grown in duplicate were incubated in the absence (control) or in the presence of either chlorophyll *a* or chlorophyllin for 1 h. Cell lysates were probed with phospho-Akt (Ser473) and phospho-ERK antibodies to determine the phosphorylation level of AKT and ERK2, respectively. Immunoblotting with ERK1/2 and p120 RasGAP antibodies was used to confirm equal protein loading.

Since chlorophyllin was reported to inhibit the proliferation of MCF-7 breast carcinoma cells by deactivating ERK [28], as well as by downregulating the PI3K/Akt signaling pathway [29], we tested whether this antiproliferative mechanism may also have some role in pancreatic cancer cells. However, the exposure of human PaTu-8902 pancreatic cancer cells to either chlorophyll *a* or chlorophyllin (50 *μ*mol/L) for 1 h did not lead to any significant changes in phosphorylation and thus in the activation of AKT or ERK (Figure 7).

### 3.6. The Effect of Chlorophylls on Viability of Human Pancreatic Cancer Xenotransplanted to Mice

**Figure 8:**
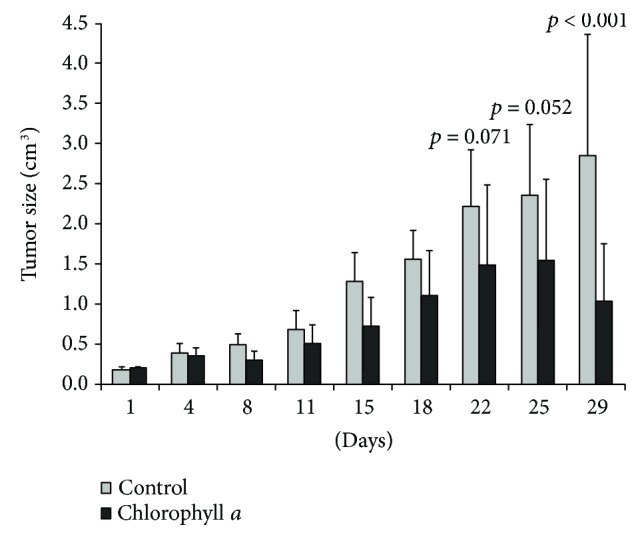
The effect of chlorophylls on growth and proliferation in human pancreatic cancer xenotransplanted to mice. Oral administration of chlorophyll *a* (1.5 mg/kg b.wt./day), *n* = 6 in each group. Group mean differences in tumor size were measured by RM ANOVA with Holm-Šidák post hoc testing.

Since chlorophylls significantly reduced the viability of pancreatic cancer cells, we tested the effect of chlorophyll *a* on tumor growth in an *in vivo* study on athymic mice xenotransplanted subcutaneously with PaTu-8902 human pancreatic cancer cells. A daily oral treatment of chlorophyll *a* led to a substantial decrease in tumor size in treated animals (Figure 8). It is of note that after a month of treatment, the pancreatic tumors were approximately one-third the size of those treated with placebo (1.04 ± 0.8 versus 2.86 ± 1.6 cm^3^, *p* < 0.001 (Figure 8)) indicating that even oral administration of the relatively poorly soluble chlorophyll *a* is a very efficient approach to prevent the progression of this malignant cancer.

## 4. Discussion

Chlorophylls, the most abundant pigments on Earth which are intrinsically incorporated into the human food chain, represent natural compounds with a powerful biological potency. Although their potentially beneficial effects on human health had already been reported in the first half of the 20th century [30] and if one also takes into account their abundance, the amount of convincing experimental and clinical data on this topic is surprisingly scarce. It is generally believed that the chemopreventive effects of chlorophylls are most likely due to their potential to trap carcinogens within the intestinal lumen [1] as demonstrated in both the Chinese [9] and Dutch clinical studies [10].

Despite long discussions about their bioavailability, chlorophylls seem to be important for their potential systemic cancer-preventive effects [31]. In fact, recent studies have reported that a biologically relevant fraction of chlorophylls (or their derivatives) is even present in peripheral tissues [4]. This was shown in a study by Gandul-Rojas and colleagues who found that the bioavailability of dephytylated chlorophyll was increased 65 times when compared to maternal pigment [32].

Although chlorophyll *a* is the major chlorophyll species present in the plant kingdom, a wide variety of other chlorophylls and their metabolites exist in various plants, algae, and animals [6] and thus find their way into the human food chain. In addition, during the plant life cycle, chlorophylls are actively metabolized within plant cells and these chlorophyll derivatives, acting as potent antioxidants, are believed to play an important role in senescence, in a manner analogous to that of bilirubin in the human body [5]. Furthermore, chlorophyll is known to be converted to pheophytin, pyropheophytin, and pheophorbide in processed vegetable foods, and after ingestion, it seems that humans may benefit from their potential biological potency [33]. Indeed, both metal-free and metallo-chlorophyll derivatives, representing dietary chlorophyll metabolites which are prevalent in both fresh and processed foods (as well as dietary supplements), have been shown to possess antioxidant, antimutagenic, and anticancer activity [34, 35]. Additionally, parts of the metabolized chlorophylls, such as phytanic acid, may exert a specific nuclear receptor-modulating activity [6, 36] through which it can contribute to the biological effects that have been observed in both experimental and clinical studies. However, it should be emphasized that the fate of chlorophylls in the human body as well as their potential biological effects still remains essentially unexplored. In fact, it is not currently known, whether the potential anticancer effects of chlorophylls are associated with native tetrapyrrolic compounds alone or their degradation products (or both), formed in the human digestive system. Furthermore, in the green plants, there might be a plethora of other biologically active compounds which contribute to or modify anticancer effects.

Nevertheless, a wide array of chemopreventive activities has been reported for chlorophyllin, the most commonly studied chlorophyll derivative, which affects multiple intracellular targets and pathways that contribute to carcinogenesis (for review, see [37]). In fact, in our *in vitro* study on human pancreatic cancer cells, we demonstrated the potent antiproliferative activities for all tested chlorophylls and found that chlorophyll *a* was the most effective. We also described these anticancer effects in our *in vivo* study of nude mice which were xenotransplanted with PaTu-8902 cells (the most sensitive human pancreatic cancer cell line in our experiments).

The antioxidant effects are among the most firmly established activities exerted by chlorophylls. For instance, it has been documented that chlorophyllin confers a higher degree of protection against free radicals in comparison to other antioxidants such as ascorbic acid and glutathione [38]. In fact, we recently observed that the antioxidant effects of the tetrapyrroles, bilirubin, and phycocyanobilin dramatically differed from those of other antioxidants. The most prominent feature of their action was the attenuation of mitochondrial ROS production [39, 40]. Here, we have shown that the same is true for chlorophylls. All chlorophylls tested not only consistently improved total peroxyl radical-scavenging activities but also suppressed the production of mitochondrial ROS and total cellular hydrogen peroxide by cancer cells, accompanied by a shift of the glutathione redox status towards a reduction.

Importantly, mitochondrial ROS govern the cellular redox signaling, and an increase in their production has been shown to be critical for tumorigenesis [41, 42]. However, cancer cells also upregulate antioxidant protection in order to maintain the balance between oxidative signaling and oxidative stress [43]. Indeed, several reports suggest that supplementation with antioxidants including N-acetylcysteine and tocopherol may, in fact, promote the progression of cancer through their protection against oxidative stress [44, 45]. However, here, we report that chlorophylls, in contrast to other antioxidants, suppressed the progression of pancreatic cancer. This effect was accompanied by attenuated expression and activity of HMOX1, a crucial component of the intrinsic cellular antioxidant system. This might really have relevance to their anticancer effects, since *HMOX1* expression has been reported to prevent the responsiveness of pancreatic cancer to cytostatic therapy [17]. It is interesting to note that this effect might be (cancer) cell-specific, since the opposite activity against HMOX1 expression was observed for chlorophyllin-treated human umbilical vein endothelial cells [46]. Our observation may not seem surprising in light of the interrelationships between chlorophyll and HMOX in plants [47]. Also, BLVRA, another important enzyme in the heme catabolic pathway, has been proposed as promoting carcinogenesis [18]. In fact, the increased expression of *BLVRA* has been demonstrated in patients with hepatocellular cancer [19], as well as in breast and lung cancer cell lines [20]. Nevertheless, in our *in vitro* studies on human pancreatic cancer cells, we did not observe any effects of chlorophylls on *BLVRA* expression.

Interestingly, we did not observe any effect of chlorophylls on glutaminolysis or reductive carboxylation, indicating that neither glycolysis nor mitochondrial respiration was affected by chlorophyll treatment [25]. Hence, it seems that the antiproliferative effects of chlorophylls are related to the suppression of ROS production and are unrelated to cell bioenergetics.

In previous studies, chlorophyllin was reported to downregulate the PI3K/Akt signaling pathway [29] and shown to inhibit the proliferation of MCF-7 breast carcinoma cells by deactivating ERK [28]. However, neither chlorophyll *a* nor chlorophyllin had any inhibitory effect on AKT or ERK phosphorylation in our pancreatic cancer cell studies.

## 5. Conclusions

Chlorophylls mediate changes of the redox status of pancreatic cancer cells which might partially be responsible for their anticancer effects and contribute to decreased incidence of cancer among consumers of green vegetables observed in clinical studies.

## Figures and Tables

**Table 1:** Sequences of the primers for the target genes.

Genes	GenBank accession number	Forward primer	Reverse primer	Fragment (bp)
*HMOX1*	NM_002133.2	GGGTGATAGAAGAGGCCAAGA	AGCTCCTGCAACTCCTCAAA	67
*BLVRA*	NM_000712.3	TCCCTCTTTGGGGAGCTTTC	GGACCCAGACTTGAAATGGAAG	180
*HPRT*	NM_000194.2	CACTGGCAAAACAATGCAGAC	GGGTCCTTTTCACCAGCAAG	92

HMOX1: heme oxygenase 1; BLVRA: biliverdin reductase; HPRT: hypoxanthine phosphoribosyltransferase 1. *HPRT* was used as the housekeeping gene. Two reference genes (*HPRT* and *GAPD*) were selected as the most stable among 4 constant genes (*HPRT*, *GAPD*, *18S RNA*, and *UBC*) based on the analyses of peripheral blood leukocytes and the DNA of 10 healthy human controls by using geNORM 3.5 (https://genorm.cmgg.be/, accessed 2009 Dec 15). Based upon similar expression levels as in target genes, *HPRT* was selected as a more appropriate control gene, compared to *GAPD*.

**Table 2:** Antiproliferative effects of chlorophylls on human pancreatic cancer cells.

Cell line	Concentration (*μ*M)	Chlorophyll *a*	Chlorophyll *b*	Chlorophyllin	Pheophytin *a*
PaTu-8902	10	**48.8**	**87.8**	94.8	**91.6**
	50	**18.6**	**65.6**	94.2	**90.2**
	125	**13.1**	**45.8**	**62.9**	**76.7**

MiaPaCa-2	10	96.2	103.4	116.5	NT
	50	**69.6**	91.4	117.1	NT
	125	**40.3**	**87.6**	110.3	NT

BxPC-3	10	97.3	107.2	104.9	NT
	50	**76.3**	54.5	98.4	NT
	125	**64.8**	75.7	84.5	NT

Data expressed as % of control, untreated cells. Bold data represent those with *p* value < 0.05 when compared to control cells. NT: not tested.

## Data Availability

All raw data supporting the results are fully available upon request from the corresponding author.
